# Green Highly Clay-Filled Polyethylene Composites as Coating Materials for Cable Industry—A New Application Route of Non-Organophilised Natural Montmorillonites in Polymeric Materials

**DOI:** 10.3390/polym12061399

**Published:** 2020-06-22

**Authors:** Stanisław Wysocki, Krzysztof Kowalczyk, Sandra Paszkiewicz, Paweł Figiel, Elżbieta Piesowicz

**Affiliations:** 1 TELE-FONIKA Kable S.A., ul. Hipolita Cegielskiego 1, 32-400 Myślenice, Poland; stanislaw.wysocki@zut.edu.pl; 2 Faculty of Chemical Technology and Engineering, West Pomeranian University of Technology in Szczecin, Piastów Ave. 42, 71-065 Szczecin, Poland; 3 Faculty of Mechanical Engineering and Mechatronics, West Pomeranian University of Technology in Szczecin, Piastów Ave. 19, 70-310 Szczecin, Poland; sandra.paszkiewicz@zut.edu.pl (S.P.); pawel.figielw@zut.edu.pl (P.F.); elzbieta.senderek@zut.edu.pl (E.P.)

**Keywords:** polymeric composite, polyethylene, cable sheath, montmorillonite, clay, flame retardancy

## Abstract

In order to develop flame retardant and relatively *green* cable coating materials, polyethylene (PE) was melt blended with 5, 7.5, or 10 wt. % of a natural calcium montmorillonite (C–Ca) pre-dispersed in EBA (ethylene-butyl acrylate copolymer), EVA (ethylene-vinyl acetate copolymer), or mEVA (EVA modified with maleic anhydride). For comparison, an organophilised montmorillonite (CW9) was tested. The main study of composites containing EBA/C–Ca, EVA/C–Ca, and mEVA/CW9 pre-dispersions revealed that both clays were not fully exfoliated in the matrix, however, C–Ca (7.5 wt. %) markedly increased limited oxygen index (LOI) from 18% O_2_ (PE) up to 22.0% O_2_. An insignificantly higher LOI value (22.2% O_2_) was noted for a sample with 10 wt. % of CW9. The fillers did not affect hardness, but spectacularly increased Young’s modulus of the compression-moulded samples (tensile strength and elongation at break values were reduced). Thermal features of the matrix were not unpredictably changed by the clays. Generally, all the tests revealed that PE filled with the chemically untreated natural C–Ca reached similar or better mechanical and thermal features than materials containing the ammonium salt-modified montmorillonite.

## 1. Introduction

Modern polymeric materials for cable sheaths should exhibit low flammability and smoke production as well as no emission of toxic gases during their combustion [1,2]. Fire resistance of thermoplastics can be improved by incorporation of appropriate additives (e.g., P-based compounds [3,4,5]) and/or throughout chemical modification of a polymeric matrix [6]. One of the most commonly used thermoplastics in the cable industry is polyethylene (PE) due to its relatively high electrical resistivity, easy processing, and lack of halogen elements, however, it is a highly flammable material and must be flame retarded. Usually, PE and other polymers are filled with aluminium trihydrate (ATH) and/or magnesium hydroxide (MDH), but their compositions exhibit relatively low mechanical properties [7,8,9]. In the last years, an application of organophilised montmorillonites (OMMTs)—as flame retardants for various polymeric materials—has attracted great interest of industrial and academic cadres [10]. Montmorillonites (MMTs) are members of the smectite clay group and exhibit a 2:1 type platelet structure. Natural MMTs (mainly sodium-type) are hydrophilic, thus they are incompatible with the hydrophobic polymers [11]. Hydrophobisation (organophilisation) and an increment of platelet–platelet gap value of a natural MMT can be achieved via exchange reactions between Na^+^ or Ca^2+^ cations of the clay and an onium salt (mainly ammonium or phosphonium cations) [11,12]. Nevertheless, the preparation of valuable OMMTs-based PE nanocomposites is difficult due to the extraordinary low polarity of the matrix (polar groups usually facilitate dissipation of the individual OMMT platelets in polymers [13,14,15]). Interestingly, the influence of OMMTs on features of polymeric materials is continually investigated (however, industrial applications of these nanofillers are limited). Besides the structure of the applied onium salts, the attention is also paid to types of dispersing agents. Due to the limited solubility of polyolefines in the commonly used solvents, OMMT dissipation in the polymers is usually realised via melt compounding methods. Correlation between OMMTs and fire resistance of LDPE was studied, for example, by Zhang and Wilkie [16]. They revealed a reduction by 30–40% of a heat release rate peak (PHRR) value of composites with a few commercial OMMTs (3 wt. %). Zhao [17] presented that the OMMT addition (up to 15 wt. %) decreased that parameter by 73% as well as positively affected mechanical parameters of PE. Lower values of PHRR of PE/OMMT (vs. PE) were also reported in [3,18]. Interestingly, samples containing 5 wt. % of a natural sodium MMT (MMT-Na) achieved slightly lower PHRR (720 kW/m^2^) than PE with 2 wt. % of an OMMT (746 kW/m^2^) while a sample with 10 wt. % of MDH reached 688 kW/m^2^ [18]. More interesting results have been reported in the latter paper: medium HRR values for PE containing 9 wt. % of (i) an OMMT, (ii) a MMT-Na, or (iii) a commercial nitrogen-phosphorus flame retardant were almost similar (198–219 kW/m^2^) [3]. Limited oxygen index (LOI) values also show that OMMTs do not reduce the flammability of polyolefins markedly more efficiently than non-organophilised MMTs. Interestingly, the studies revealed that PE/OMMT materials often contain aggregates of the clay and should be considered as microcomposites. Limited exfoliation of various OMMTs (the absence of their statistic dissipation for individual platelets) in the PE matrix have been reported in e.g., [16,19]. Only insignificant increments of interlayer spacing values—resulted from polymeric chains migration into platelet–platelet gaps (an intercalation process)—were described in these and many other papers. It shows that organophilisation of MMTs and their further dispergation in PE (using designed dispergation agents, for example, PE-g-MA [3,16,19]) do not usually allow one to produce nanocomposite-type polymeric materials with extraordinary better features than PE/MMT-Na systems. Moreover, the initial form of the clay is significantly cheaper than an OMMT, which is crucial for industrial applications. It is generally not discussed in the literature, but organophilisation processes are commonly realised via treating of aqueous MMTs-Na suspensions by an excess (than CEC) of bioactive quaternary ammonium salts. Formed by-products (mainly NaCl or NaBr) as well as unreacted excess of the bioactive modifier are removed from created OMMTs throughout washing with plenty of water. Considering the global water deficits, the non-ecological modification process of the clay—and the mentioned aggregation tendency of the OMMTs—extremely diminish the attractiveness of this (nano)fillers in comparison with the natural MMTs and other nanosized natural or synthetic platelet-type modifiers, for example, it is known that graphenes may positively affect thermal, barrier and mechanical features of many polymers [20,21].

Many research groups investigated various polymeric composites with MMTs-Na [3,18,20,22], but compositions with calcium montmorillonites (MMTs-Ca; the naturally occurring less hydrophilic MMTs) were not widely described in the literature. It should be noted that MMTs-Na (before their organophilisation) are often formed via MMTs-Ca activation/cation exchange processes using soda. Interestingly, natural MMTs deposits always consist of MMTs-Ca or MMT-Ca/MMT-Na mixtures (the latter mineral does not occur alone). Features of polystyrene filled with a MMT-Ca were analysed in [23], however, this raw clay was not tested in polyolefins. Considering the relatively positive influence of the clays on flammability limitation of a few thermoplastic polymers as well as the above-mentioned disadvantages of OMMTs, it seems to be interesting to investigate PE composites with a high content of MMTs-Ca. As noted above, this polymer is commonly used in the cable industry, thus the development of non-sophisticated, cheap, and halogen-free compositions as well as forming methods of the flame retardant PE-based materials is one of the most valuable challenges. In this paper, a commercial raw MMT-Ca was tested as a *green* component of a highly-filled LLDPE matrix. The clay was pre-dispersed in the known exfoliation agents for many OMMTs, i.e., EBA, EVA, or mEVA (EVA grafted with maleic anhydride). For comparison, a commercial OMMT was tested. The highest preferred concentration value of commercially available OMMTs in different polymeric systems (5 wt. %) was adopted in this study as the lowest content of the tested fillers in PE composites.

## 2. Materials and Methods

### 2.1. Materials

A linear low-density polyethylene (PE) (LLDPE 1004AY; ExxonMobil, Irving, TX, USA) was used as a polymeric matrix of the MMT-based composites. The following ethylene copolymers were used as dispersing agents for the tested aluminosilicates:

- An ethylene-butyl acrylate copolymer (EBA) with melting temperature ca. 95 °C and density 0.92 g/cm^3^ (Lucalen A2700M; LyondellBasell, Rotterdam, The Netherlands);
- An ethylene-vinyl acetate copolymer (EVA) with melting temp. ca. 73 °C, density 0.95 g/cm^3^ and 28 wt. % of VAc (Elvax 265; DuPont, Wilmington, DE, USA);
- An ethylene-vinyl acetate copolymer modified with maleic anhydride (m-EVA) with melting temp. ca. 71 °C and density 0.96 g/cm^3^ (Fusabond C250; DuPont, Wilmington, DE, USA).

The PE and the above-mentioned modifiers were compounded with the montmorillonites (MMTs):

- A powdered natural calcium montmorillonite (C–Ca) with an average particle size (d_50_) of 10 μm (Cloisite Ca++; BYK-Chemie, Wesel, Germany);
- A powdered montmorillonite (CW9) organophilised with dimethyl benzyl hydrogenated tallow ammonium salt and with an average particle size (d_50_) of 20 μm (Dellite CW9; Laviosa Chimica Mineraria, Livorno, Italy).

### 2.2. Composites Preparation

The MMT-based composites were prepared via a two-step melt compounding method. In the beginning, 60 wt. % parts of the montmorillonite (C–Ca or CW9) was extruded with 40 wt. % parts of the dispersing agent (EBA, EVA, or m-EVA) using a conventional co-rotating twin-screw extruder (Prism EuroLab 16; Thermo Electron Corporation, Waltham, MA, USA) equipped with a 2 mm hole die. The extrusion process was realised at the screw speed of 100 rpm and the temperature (from a components inlet to a nozzle) of 110/130/150/160/170/180/180/180/180/180 °C. The line of a polymeric composition was cooled down using water and cut into 3-mm length pellets. Then the pellets were dried at 70 °C for 72 h. After drying, the prepared MMT pre-dispersions (i.e., EBA/C–Ca, EBA/CW9, EVA/C–Ca, EVA/CW9, m-EVA/C–Ca and m-EVA/CW9) were extruded with PE at the same conditions. After drying, extruded PE composite pellets (containing 5, 7.5 or 10 wt. % of the MMTs and respective amounts of the dispersing agents) were compression-moulded at 190 °C by means of a heated hydraulic platen press (Remi-plast, Czerwonak, Poland). Dimensions of the samples were 110 mm × 110 mm × 2 mm. For comparison, samples containing only PE were prepared.

### 2.3. Methods

Wide-angle X-ray diffraction analyses (XRD) of the powdered MMTs and MMT-filled composites were realised using the PW 3040/6 X’Pert Pro diffractometer (CuKα, 3–60° 2θ; PANalytical, Almelo, The Netherlands) equipped with the X’Pert High Score 2.2a analysis software.

Thermal analyses of the composites (during their cooling and heating) were carried out using the differential scanning calorimeter DSC Q100 (TA Instruments, New Castle, DE, USA). The tests were performed as follow: a sample was initially heated to 190 °C (the temperature of the compression-moulding process) and kept at this temperature for 1 min; then (the main test) it was cooled down to −90 °C (10 °C/min) and subsequently heated to 200 °C (10 °C/min). As a result, two curves were registered (cooling from 190 to −90 °C and heating from −90 to 200 °C).

Tensile tests of the MMT-based PE composites were performed by means of Instron 3360 machine (Instron, Norwood, MA, USA) using dumbbell-shaped specimens. Tensile strength (TS), elongation at break (EaB), and Young’s modulus (E) values were determined according to PN-EN ISO 60811-2-1. Five specimens of each sample were analysed, and mean values with standard deviations were calculated. The tests were realised at room temperature (50% RH). The Shore D-type hardness of the composites was measured using the Zwick 3100 apparatus (ZwickRoell, Ulm, Germany).

Limited oxygen index (LOI) values of the composites were measured acc. to the ISO 4589 standard using the OIM apparatus (Concept Equipment, Rustington, UK). Samples were stored at RT (50% RH) for 88 h before testing. Additionally, thermostability of the composites, i.e., the temperature of 10 (*T*_10_) and 50% mass loss (*T*_50_) as well as a calcination residue were measured using the thermogravimetric analyzer Q5000IR (25–900 °C, 10 °C/min, air atmosphere; TA Instruments, New Castle, DE, USA).

## 3. Results

### 3.1. Selection of MMT Dispersing Agents

**Figure 1:**
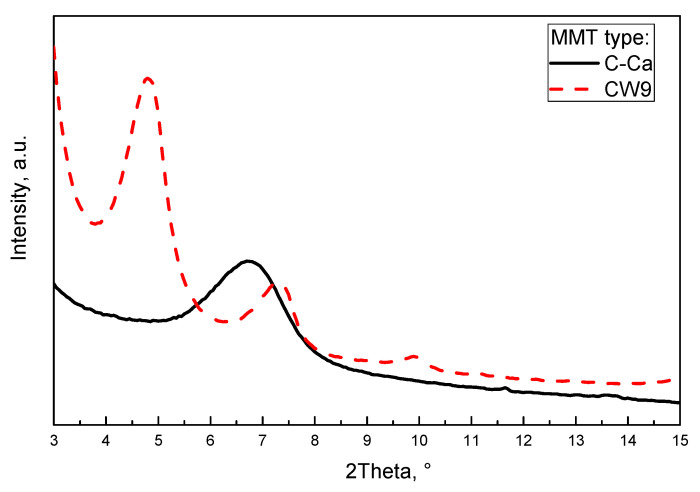
X-ray diffraction (XRD) patterns for powdered MMTs.

At the beginning of the main study of highly MMT-filled PE-based composites, a selection of the most efficient dispersing agent (for the each MMT) has been realised. In the cable industry, the crucial mechanical parameters of polymeric shields are tensile strength (TS), elongation at break (EaB), and Young’s modulus (E) values as well as their flammability (mainly LOI). Other mechanical features, such as impact or bending strength are much less important according to the commonly applied standards. Due to this fact, the selection of the MMT dispersing agents from EBA, EVA and m-EVA copolymers was realised via investigation of their influence on tensile test results of PE composites containing 5 wt. % of the unmodified (C–Ca) or organophilised montmorillonite (CW9). As can be seen in Table 1, the applied dispersing agents and MMTs significantly affect the above-mentioned mechanical parameters of the compression-moulded samples. Generally, all the MMT-filled systems reached significantly lower TS (9.7–14.9 MPa) than the reference material (PE, 15.7 MPa). Only the composition containing the m-EVA/CW9 pre-dispersion (PmEVA/CW-5) exhibited a similar value of this parameter (15.5 MPa) in relation to the unfilled PE. It should be noted that TS values for the samples with the EBA/C–Ca or EVA/C–Ca pre-dispersions (14.7 MPa for PEBA/Ca-5 and 14.9 MPa for PEVA/Ca-5) were markedly higher in comparison with the systems containing EBA/CW9 (10.6 MPa) or EVA/CW9 (9.7 MPa). This relation was reversed for the samples containing the m-EVA agent, and PmEVA/CW-5 reached a quite higher TS (15.5 MPa) than PmEVA/Ca-5 (10.9 MPa). It shows that platelets (or aggregates) of the organophilised montmorillonite (CW9) were more effectively dispersed in the PE matrix contained the EVA copolymer enriched with maleic anhydride (mEVA) (as it was noted in [24]). On the other hand, the applied unmodified copolymers/dispersing agents (EBA, EVA) more positively affected the mechanical parameters of the C–Ca-filled composites. However, the m-EVA additive is a relatively more polar and hydrophilic component than EBA and EVA (thus, it should more effectively dissipate/exfoliate the polar MMT such as C–Ca), a grafting reaction of maleic anhydride increases coil size and reduces the mobility of this dispersing agent. In this case, m-EVA exhibits higher affinity for CW9 (due to a higher maximal initial interlayer spacing value; a higher TS for the sample with the mEVA/CW9 pre-dispersion) than for the C–Ca filler (a lower initial interlayer spacing; a lower TS for mEVA/C–Ca-based composites). As can be seen in Figure 1 (XRD patterns for the powdered MMTs), the former clay was characterised by the main interlayer gap of ca. 36.8 Å (for 4.8° 2θ and *n* = 2 as well as 36.3 Å for 7.3° 2θ and *n* = 3 [25]) while the interlayer spacing value for the C–Ca filler was only 13.2 Å (6.7° 2θ, *n* = 1). It is known that even limited intercalation of MMTs positively (or neutrally) affects many mechanical features of polymeric (nano)composites [26,27].

As can be seen in Table 1, the influence of MMT pre-dispersion addition on EaB values of the composites is generally similar to the one observed for TS; the highest elongation values were registered for the reference sample (PE, 785%) and the sample with the m-EVA/CW9 pre-dispersion (PmEVA/CW-5, 769%). Nevertheless, quite high values of this parameter were also observed for the samples with the natural montmorillonite and the other dispersing agents (EBA, EVA): PEBA/Ca-5 (763%) and PEVA/Ca-5 (711%). Interestingly, EBA (used for pre-dispergation of both MMT types) extraordinarily affected the E parameter of the prepared composites (Table 1). The samples containing EVA (279 MPa for PEVA/Ca-5 and 284 MPa for PEVA/CW-5) as well as the m-EVA-based composites (221 MPa for PmEVA/Ca-5 and 347 MPa for PmEVA/CW-5) reached similar values of this parameter (in relation to the reference sample; 273 MPa), but the EBA dispersing agent increased the modulus up to ca. 4.7 GPa (the sample with EBA/CW9) and 9.0 GPa (EBA/C–Ca). It is noteworthy that a similarly high increment of the E parameter (for PE/MMT-type composites) has not yet been described in the literature. It should be noted that this phenomenon does not directly result from the EBA presence in the polymeric matrix; the E value for a PEBA system (the PE/EBA composition without a MMT; data not presented) was only 0.9 GPa while PE/EVA and PE/mEVA reached 1.3 GPa and 2.7 GPa, respectively. It shows that the MMT addition caused the spectacular E increment recorded for PEBA/Ca-5 and PEBA/CW-5. As mentioned above, the former sample (the higher E value) reached also higher TS (14.7 MPA) and EaB (763%) than the latter one (10.6 MPa, 578%), however, these mechanical parameters values (except for E) were not higher in relation to the reference sample (PE, 15.7 MPa, 785%).

Requirements for halogen-free polymeric compounds designed for cable jackets are described in specific EN standards. Generally, TS of these materials should be at least 9 MPa (EaB ≥ 125%) [28]. It shows that all the prepared samples with the natural calcium MMT (or CW9) fulfil the mentioned requirements for the industrial application. Nevertheless, for further experiments only materials with the highest TS have been chosen. In this case, highly MMT-filled PE composites (7.5 or 10 wt. % of the clays) were prepared using the EBA/C–Ca, EVA/C–Ca as well as m-EVA/CW9 pre-dispersions.

### 3.2. Properties of Highly MMT-Filled PE Composites

**Figure 2:**
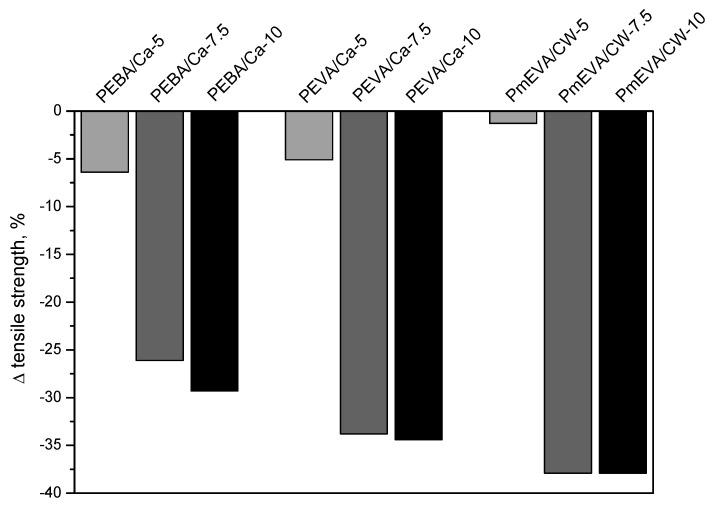
Influence of a MMT pre-dispersion type and MMT content (5, 7.5 or 10 wt. %) on tensile strength values of PE composites.

**Figure 3:**
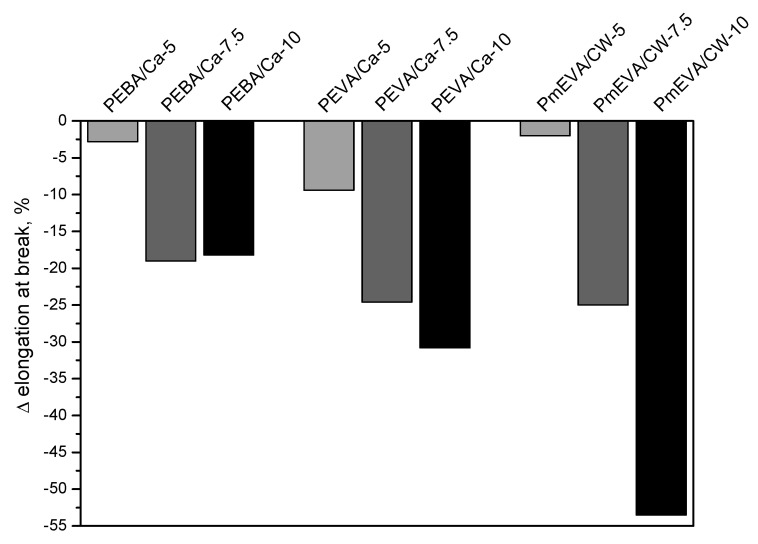
Influence of a MMT pre-dispersion type and MMT content (5, 7.5 or 10 wt. %) on elongation at break value of PE composites.

**Figure 4:**
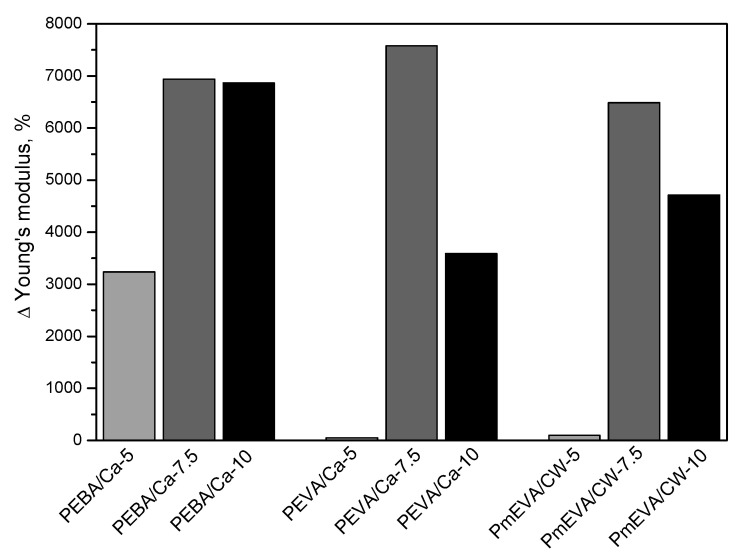
Influence of a MMT pre-dispersion type and MMT content (5, 7.5 or 10 wt. %) on Young’s modulus value of PE composites.

The mechanical parameters registered during tensile tests of PE composites with the higher concentration of the selected MMT pre-dispersions (EBA/C–Ca, EVA/C–Ca and m-EVA/CW9) are shown in Table 1. Additionally, percentage changes of the mentioned features (in relation to the reference PE sample) were graphically presented in Figure 2 (tensile strength—TS), Figure 3 (elongation at break—EaB) and Figure 4 (Young’s modulus—E). As can be seen, the increment of MMTs content (different dispersing agents) resulted in a significant reduction of TS and EaB values. The largest decrement of the former parameter was noted for materials with the mEVA/CW9 pre-dispersion (−37% for PmEVA/CW-7.5 and PmEVA/CW-10 vs. PE). In the case of EaB, it was markedly decreased for PEVA/Ca-7.5 and PmEVA/CW-7.5 (ca. −25%; systems with 7.5 wt. % of a MMT) and for PmEVA/CW-10 (−54%; systems with 10 wt. % of a MMT). It is known that addition of different MMT types may restrain slippage movement of polymeric chains during deformation of many polymeric materials and decrease their EaB values [29,30,31].

**Figure 5:**
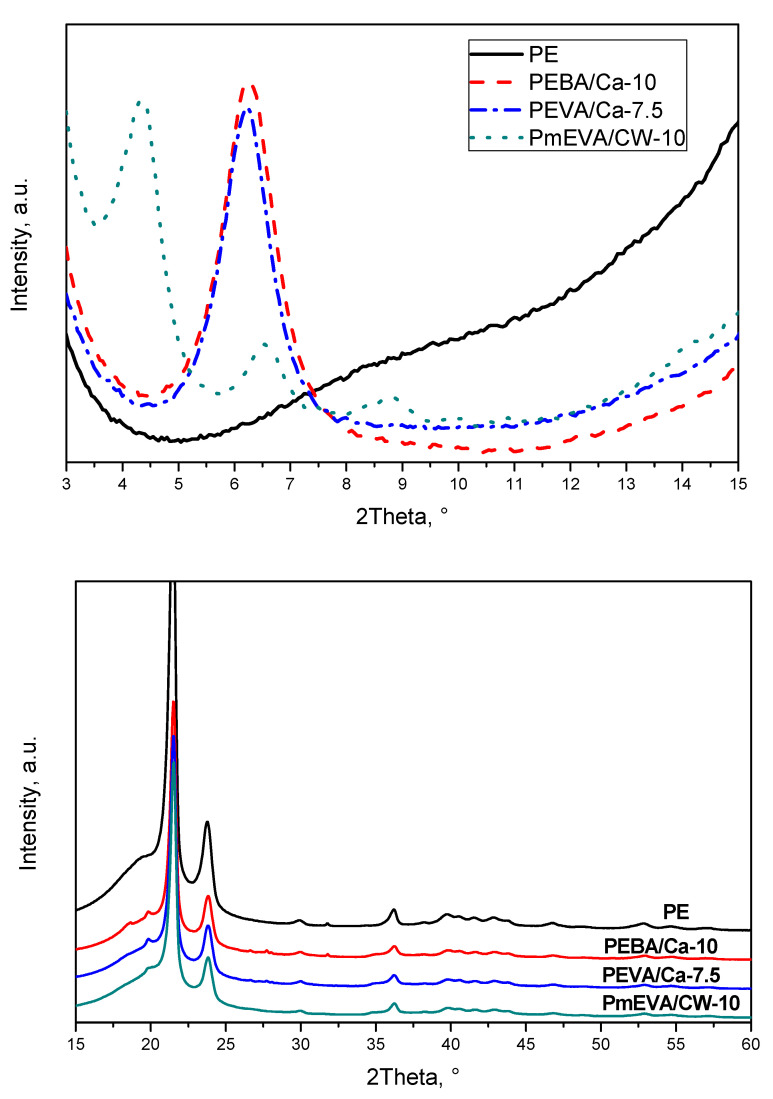
XRD patterns for selected MMT-based PE composites.

Considering the presented results, it seems that all the MMT pre-dispersions with vinyl acetate copolymer-type dispersing agents (i.e., EVA or m-EVA) most negatively affected both mechanical parameters of the composites than the EBA-based systems. Moreover, samples with the organophilised aluminosilicate (CW9) reached usually lower TS and EaB values in comparison with the relevant materials containing 7.5 or 10 wt. % of the natural montmorillonite. Nevertheless, it should be reminded that the sample with the lowest mEVA/CW9 pre-dispersion content (PmEVA/CW-5) reached higher TS and EaB values in relation to PEBA/Ca-5 and PEVA/Ca-5 (Figure 2, Figure 3). Arguably, the observed relation was caused by different MMT penetration efficiency of the dispersing agents as well as by a particle size value of the applied fillers. The XRD analyses of selected composites (with the highest LOI values; see the text below) revealed slight intercalation of the clays aggregates: interlayer spacing increased to ca. 14.1 Å (for all the composites with C–Ca) and 40.7 Å for the CW9-based composite (three peaks at 4.35° 2θ and *n* = 2, at 6.55° 2θ and *n* = 3, at 8.8° 2θ and *n* = 4; Figure 5). It shows that the tested ethylene copolymers (EBA, EVA and mEVA)—described in the literature as relatively effective dispersing agents for various OMMTs [18,21,32]—do not allow to prepare nanocomposite materials with fully and statistically dissipated clay platelets in the polymeric matrix. Nevertheless, due to the relative high maximal (initial) interlayer spacing value of CW9 (ca. 36.8 Å; Figure 1), a part of this filler was arguably exfoliated in the sample. In this case, PmEVA/CW-type materials should contain a mixture of separated platelets and their intercalated aggregates (d = 40.7 Å). It was widely described in the literature that exfoliated OMMTs positively affect mechanical parameters of PE and other polymers while their aggregates may reduce these features. In the authors’ opinion, the positive influence of the dissipated CW9 platelets (on TS values) plays a crucial role in the low-filled material (PmEVA/CW-5)—this sample reached higher TS than the other samples with 5 wt. % of C–Ca. On the other hand, the TS values of the highly CW9-filled materials (PmEVA/CW-7.5 and PmEVA/CW-10) mainly resulted from the clay aggregates presence—these samples exhibited much lower TS than the other samples with C–Ca. Moreover, the particle size value of the powdered C–Ca was much lower (ca. 10 μm) in relation to CW9 (20 μm), thus aggregates of the raw aluminosilicate should be better dispersed in the polymeric matrix. A negative impact of the mEVA addition on the TS and EaB parameters (caused by a potential molecular weight reduction of an EVA copolymer during a maleic anhydride grafting process) is rather low. An OMMT-free PE/mEVA sample (data not presented) reached relative high values of these parameters, i.e., 14.1 MPa and 590% (the comparable PmEVA/CW-10 sample exhibited 9.9 MPa and 365%). Nevertheless, it should be noted that even PE nanocomposites with a few types of relatively well dispersed organophilised montmorillonites reached lower TS than the unmodified polymer. A similar observation was done for PE-based systems with MMT-Na clays as well [33,34].

Interesting results were registered for Young’s modulus of the prepared composites. Generally, the higher clays concentration (7.5 wt. %) significantly increased E value in relation to the reference sample and the materials containing only 5 wt. % of the fillers (Table 1, Figure 4). Nevertheless, the composites with 10 wt. % of the aluminosilicates and the vinyl acetate-based dispersing agents (PEVA/Ca-10 and PmEVA/CW-10) were markedly less rigid than PEVA/Ca-7.5 and PmEVA/CW-7.5. This relation was not observed for the materials with the EBA/C–Ca pre-dispersion (E values were quite similar for PEBA/Ca-7.5 and PEBA/Ca-10). Taking into consideration that the E reduction was registered for both composites containing the similar dispersing agents, i.e., EVA and mEVA (but different MMT types), it could be claimed that the mentioned phenomenon directly resulted from the higher clay content. Probably, too high concentration of the vinyl acetate copolymers reduces E of the composites, however, values of this parameter for PEVA/Ca-10 (ca. 19 GPa) and PmEVA/CW-10 (13 GPa) were dramatically higher than for the reference sample (PE, 0.3 GPa).

As can be seen in Table 2, the hardness of all the composites was similar or insignificantly lower (−2° ShD) in comparison with the PE sample (38° ShD). The mentioned differences may be caused by the introduction of the dispersing agents (EBA, EVA and m-EVA) It is generally known that butyl acrylate or vinyl acetate comonomers may upgrade rubber behaviour of polymeric materials [35,36]. On the other hand, hardness and other mechanical features (TS, EaB and E) of polymers are often affected by their crystallinity, whereas incorporation of aluminosilicates may disturb the ordered structures of polymeric chains [37]. Nevertheless, the XRD analyses of the prepared materials (15–60° 2θ, Figure 5) revealed no significant influence of both clay types on the crystallinity of the PE matrix. The recorded WAXS patterns for the reference sample and the selected composites were quite similar (except for a small peak at ca. 18.6° 2θ characteristic for EBA dispersing agent [38]).

**Figure 6:**
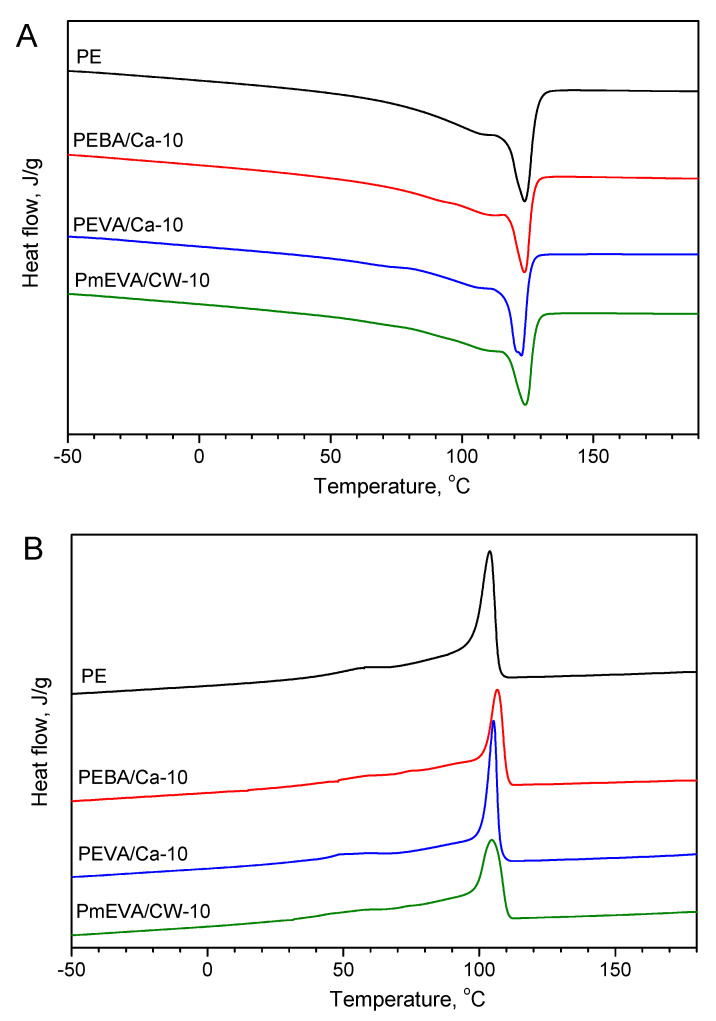
DSC curves at heating (**A**) and cooling modes (**B**) for PE composites with the highest MMT content.

Results of the DSC analyses (Table 3, Figure 6) shown a slight influence of the clay addition on the total enthalpy of melting and crystallisation processes of the composites. Generally, the enthalpy values decrease with increasing content of the pre-dispersions and they were mostly reduced for the samples with EVA/C–Ca (from 116.1 to 87.9 J/g during heating and from 110.0 to 87.4 J/g during cooling of PEVA/Ca-10). Arguably, the noted reduction was caused by the incorporation of the infusible inorganic filler (C–Ca, CW9) with a relatively low heat capacity (ca. 0.98 J/g at 400 K for MMT-Ca [39]), and the semi-crystalline dispersing agents (EBA, EVA or mEVA). On the other hand, onset temperature values as well as peak temperature values for the crystallisation process of PE and all the composites were quite similar (111–114 °C and 104–107 °C, respectively). It seems that the tested clays do not markedly affect a nucleation phenomenon of the PE matrix [19,40]. It correlates with the above-mentioned WAXS data.

One of the most important parameters of polymeric compounds used as cables sheaths is limited oxygen index (LOI) representing oxygen concentration value in the atmosphere needed to continuous burning of the materials. In the case of LOI < 21% O_2_, a material burns easily in the air after removing an ignition source [41]. LOI varies for different materials and reached 18.0% O_2_ for the tested reference PE sample (the same value was presented in [42]). As can be seen in Table 2, all the prepared composites exhibited higher LOI values (21.0–22.2% O_2_) in relation to the unmodified sample, thus they are classified as self-extinguishing materials (their combustion cannot be sustained at ambient temperature without an external energy contribution [42]).

**Figure 7:**
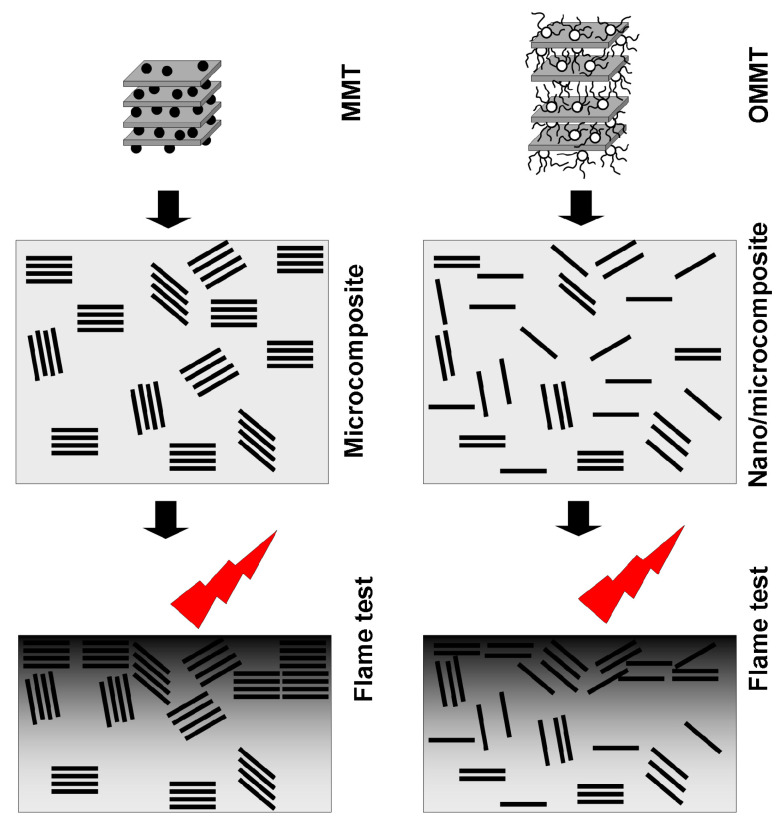
Schematic structures of PE nano/microcomposites containing MMT (C–Ca) or organophilised montmorillonite (OMMT) (CW9) (before and after LOI tests).

Generally, the LOI value increases with increasing clay content in the systems, but the highest increment (+3.5% O_2_ vs. PE) was registered for the materials with the lowest clays concentration (5 wt. %). This phenomenon was mainly observed for the samples with the clay pre-dispersions based on EVA or m-EVA (due to a higher fire resistance of these copolymers in comparison with EBA [41,43]). On the other hand, the highest LOI value was noted for PmEVA/CW-10, arguably, it resulted from modification of an EVA copolymer with maleic anhydride. It should be noted that the LOI results (recorded for the composites containing C–Ca) are markedly higher than these presented in many papers describing MMT-Na-based PE (nano)composites. For example, de Oliveira et al. increased the LOI value of PE from 18.3% O_2_ only to 19.0% O_2_ by its compounding with a Brazilian clay (pre-dispersed in a PE-g-MA component). Interestingly, a sample containing 6 wt. % of this clay reached slightly higher LOI than a material with the same concentration of an OMMT filler (18.7% O_2_) (similar values of this parameter were noted for samples with either 6 wt. % of the MMT-Na or 9 wt. % of the OMMT clay). Moreover, as mentioned in the introduction section, the incorporation of the commercial nitrogen–phosphorus-type fire retardant (6–9 wt. %) did not markedly increase the LOI of the polymer [3]. In another study, the addition of a MMT-Na clay (5 wt. %) reduced LOI of PE from 19.9% O_2_ to 19.1% O_2_ while an OMMT filler increased this feature only to 20.6% O_2_ (and to 20.7% O_2_ after incorporation of an EVA copolymer). It is noteworthy that the similar LOI value (20.6% O_2_) was registered for a PE composite filled with 20 wt. % of magnesium hydroxide [18]. It is generally supposed that the flammability of various OMMT-based polymeric materials is reduced by the formation of thermally insulating surface layers containing clay nanoparticles. These coating act as a physical barrier for volatile products, which are generated during the material combustion and feed the flame [24,44]. Considering limited thermal stability of commonly used ammonium modifiers (their degradation begins at 150–200 °C by the Hofmann elimination [24]) as well as limited exfoliation of many OMMTs [3,16,17,18], it can be claimed that the formed ceramic layers contain individual platelets (in a lower amount) and their aggregates (in a higher amount). The thermally degraded OMMTs exist in the charred layers in protonated forms [24]. In the case of the tested PE/C–Ca composites, the layers should contain MMT-Ca platelets aggregates (Figure 7). Thus, the registered LOI values for samples with either MMT-Ca or CW9 were quite similar.

**Figure 8:**
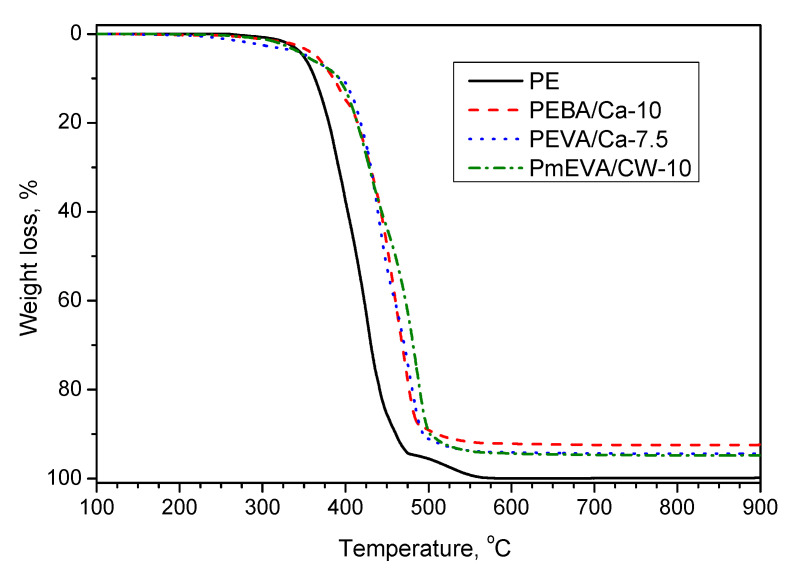
Thermogravimetric curves for selected MMT-based PE composites.

It should be noted that the recorded LOI values partly correlate with the thermogravimetric stability of the composites in the oxygen atmosphere (Table 2; exemplary curves are presented in Figure 8). Generally, temperature values at 10% (*T*_10_) and 50% mass loss (*T*_50_) of the MMT-based materials were much higher in comparison with the reference PE sample. In the case of the compositions containing 5–10 wt. % of the clays, the former parameter increased from 363 °C (PE) to 421 °C while *T*_50_ increased from 413 to 461 °C. The samples with CW9 exhibited the lowest mass loss velocity (because ammonium-type modifiers may catch free radicals created during PE thermal decay [45,46]), however, *T*_50_ values for C–Ca-based composites were quite high as well. It should be mentioned that the samples containing the highest amounts of EVA/C–Ca or mEVA/CW9 pre-dispersions were characterised by a lower thermostability than PEVA/Ca-7.5 and PmEVA/CW-7.5, respectively. Considering the different types of the applied MMTs, that phenomenon probably resulted from the presence of the vinyl acetate-type copolymers, i.e., EVA and mEVA (that effect was not observed for the samples with EBA). It shows some interaction between these copolymers and aluminosilicates during their heating. Interestingly, the efficiency of a calcination residue (CR) creation process does not linearly depend on clay concentration. In the case of samples with 7.5 wt. % of CW9, the CR value (at 900 °C) was 4.4 wt. %, while—in relation to the sample with 5 wt. % of this MMT (3.1 wt. %)—they should reach ca. 4.7 wt. %. It represents 94% efficiency of char creation during heating of PmEVA/CW-7.5. Furthermore, this efficiency was lower for PmEVA/CW-10 and reached only 84% (vs. PmEVA/CW-5). Thus, the PE composites with a higher content of the organophilised clay were characterised by a smaller CR (in relation to the applied MMT concentration). On the other hand, the higher C–Ca concentration, the higher efficiency of the CR formation process. It reached 81% (PEVA/Ca-7.5) and 86% (PEVA/Ca-10 vs. PEVA/Ca-5) as well as 82% (PEBA/Ca-7.5) and 88% (PEBA/Ca-10 vs. PEBA/Ca-5), but the total CR values for the samples with EVA/C–Ca were generally higher (4.6–7.9 wt. %) than for the EBA/C–Ca-based systems (4.3–7.6 wt. %). It confirms the positive influence of the vinyl acetate-based dispersing agent (in comparison with EBA) on thermal features of PE filled with the MMT-Ca clay.

In summary, considering that the PEVA/Ca-7.5 material exhibits higher (vs. the other materials with the unmodified MMT) or similar LOI values (vs. the sample with the same content of the OMMT, i.e., PmEVA/CW-7.5), it can be claimed that this composition is the most interesting among the other tested in this study. The mentioned system is also characterised by: (i) the relatively higher (vs. the other samples with C–Ca) or slightly lower (vs. the CW9-based samples) values of *T*_10_ and *T*_50_, (ii) the similar hardness with the reference and the other MMT-based composites, (iii) the similar *T*_H-max_ and *T*_C-max_ values (i.e., peak temperatures of melting and crystallisation processes, respectively) with the unmodified PE and (iv) the relatively higher TS, EaB and E values than materials with the same (or higher) content of CW9. By fulfilling the requirements presented in [28], the PEVA/Ca-7.5 composite seems to be outstandingly interesting (and relatively cheap) green material for further study of preparation of halogen-free flame retardant cable sheaths.

## 4. Conclusions

Polyethylene was modified with 5, 7.5 or 10 wt. % of a natural calcium montmorillonite (C–Ca) pre-dispersed in EBA (ethylene-butyl acrylate copolymer), EVA (ethylene-vinyl acetate copolymer) or mEVA (EVA modified with maleic anhydride). For comparison, a commercial organophilised montmorillonite (CW9) was tested as well. The compounds have been prepared by a convenient melt compounding technique using a co-rotating twin-screw extruder. At the beginning of the study, the efficiency of the dispersing agents was assessed considering PE composites with the lowest MMT content. As a result, for the further tests the materials with the higher concentrations of C–Ca (in EBA or EVA) and CW9 (in mEVA) were chosen. Taking into consideration the analyses results for the prepared materials with the aluminosilicate masterbatches, the following main conclusions can be drawn:

- All of the tested aluminosilicates were not fully exfoliated in the PE matrix, however, slight intercalation of the clays was observed by the XRD technique.
- C–Ca and CW9 pre-dispersions did not affect the crystallinity of PE matrix (the XRD analyses) as well as did not unpredictably change thermal features of the material during thermal processing (DSC analyses at a heating/cooling mode).
- The incorporation of the MMT pre-dispersion (all of the tested types) into PE reduced tensile strength and elongation at break of the compression-moulded samples. On the other hand, both fillers spectacularly increased Young’s modulus values of the composites, however, the highest doses of the EVA/C–Ca or mEVA/CW9 pre-dispersions diminished this phenomenon. The hardness of the reference PE material was not markedly affected by the fillers.
- The C–Ca addition markedly increased limited oxygen index (LOI) value from 18% O_2_ (PE) up to 22.0% O_2_ (the PEVA/Ca-7.5 composite with the EVA/C–Ca pre-dispersion, 7.5 wt. % of the natural clay). An insignificantly higher LOI value (22.2% O_2_) was noted for the system with 10 wt. % of CW9. Materials filled with this clay exhibited the highest temperature values at 10% and 50% mass loss during their heating in the air. Nevertheless, the amount of calcination residue (at 900 °C) nonlinearly increased with the increasing initial concentration of the C–Ca filler (in relation to the sample containing 5 wt. % of this clay). In the case of the CW9 additive, this relation was reversed (the higher filler content, the lower efficiency of the calcination residue formation process).
- Due to the medium content of the unmodified montmorillonite, the high LOI value as well as the acceptable mechanical features, the PEVA/Ca-7.5 composite seems to be the most interesting material for further study.

## Figures and Tables

**Table 1:** Mechanical features of polyethylene (PE)composites with different montmorillonites (MMTs) and dispersing agents.

Sample Symbol	Dispersing Agent	MMT Type	MMT Content ^a^	Tensile Strength (MPa)	Elongation at Break (%)	Young’s Modulus (MPa)
PE	—	—	0	15.7 ± 0.5	785 ± 85	273 ± 55
PEBA/CW-5	EBA	CW9	5	10.6 ± 0.8	578 ± 84	4677 ± 350
PEBA/Ca-5	EBA	C–Ca	5	14.7 ± 2.4	763 ± 58	9093 ± 681
PEBA/Ca-7.5	EBA	C–Ca	7.5	11.6 ± 1.7	636 ± 127	19,207 ± 195
PEBA/Ca-10	EBA	C–Ca	10	11.1 ± 1.3	642 ± 23	19,022 ± 981
PEVA/CW-5	EVA	CW9	5	9.7 ± 0.3	116 ± 75	284 ± 189
PEVA/Ca-5	EVA	C–Ca	5	14.9 ± 0.4	711 ± 26	279 ± 44
PEVA/Ca-7.5	EVA	C–Ca	7.5	10.4 ± 0.7	592 ± 83	20,964 ± 410
PEVA/Ca-10	EVA	C–Ca	10	10.3 ± 1.1	543 ± 232	10,068 ± 792
PmEVA/Ca-5	m-EVA	C–Ca	5	10.9 ± 0.9	606 ± 16	221 ± 27
PmEVA/CW-5	m-EVA	CW9	5	15.5 ± 0.5	769 ± 46	347 ± 86
PmEVA/CW-7.5	m-EVA	CW9	7.5	9.9 ± 0.3	589 ± 122	17,982 ± 412
PmEVA/CW-10	m-EVA	CW9	10	9.9 ± 0.2	365 ± 130	13,139 ± 625

^a^ wt. %.

**Table 2:** Hardness and thermal properties (TGA and limited oxygen index (LOI)) of PE composites with selected MMTs and dispersing agents.

Sample Symbol	Hardness (°ShD)	TGA Results			Oxygen Index (% O_2_)
		*T*_10_ (°C) ^a^	*T*_50_ (°C) ^a^	CR (wt. %) ^b^	
PE	38 ± 2	363	413	0	18.0
PEBA/Ca-5	36 ± 3	354	434	4.3	21.0
PEBA/Ca-7.5	38 ± 2	365	448	5.3	21.5
PEBA/Ca-10	36 ± 2	386	452	7.6	21.5
PEVA/Ca-5	37 ± 1	378	441	4.6	21.5
PEVA/Ca-7.5	37 ± 2	393	448	5.6	22.0
PEVA/Ca-10	38± 2	361	435	7.9	21.5
PmEVA/CW-5	38 ± 2	404	456	3.1	21.5
PmEVA/CW-7.5	36 ± 1	421	461	4.4	21.8
PmEVA/CW-10	36 ± 2	392	460	5.2	22.2

^a^ Temperature at 10 or 50% mass loss; ^b^ Calcination residue (at 900 °C).

**Table 3:** Thermal properties (differential scanning calorimeter (DSC)) of PE composites with selected MMTs and dispersing agents.

Sample Symbol	Heating			Cooling		
	*T*_H-0_ (°C) ^a^	*T*_H-max_ (°C) ^b^	Q_H_ (J/g) ^c^	*T*_C-0_ (°C) ^a^	*T*_C-max_ (°C) ^b^	Q_C_ (J/g) ^c^
PE	39.7	123.9	116.1	110.8	103.9	110.0
PEBA/Ca-5	42.1	123.4	107.2	112.2	106.0	103.7
PEBA/Ca-7.5	42.9	123.6	99.9	112.7	106.3	96.3
PEBA/Ca-10	38.6	123.8	95.7	113.5	106.6	89.7
PEVA/Ca-5	47.4	123.1	95.0	111.6	105.1	98.3
PEVA/Ca-7.5	46.4	123.8	92.2	111.4	104.3	91.1
PEVA/Ca-10	41.4	122.7	87.9	111.1	105.3	87.4
PmEVA/CW-5	45.8	123.5	101.5	114.3	103.9	98.0
PmEVA/CW-7.5	41.3	123.7	97.0	113.5	103.6	93.8
PmEVA/CW-10	38.6	124.2	95.6	112.2	104.6	93.2

^a^ Onset temperature of a melting (*T*_H-0_) or crystallisation process (*T*_C-0_); ^b^ Peak temperature of the melting (*T*_H-max_) or crystallisation process (*T*_C-max_); ^c^ Total enthalpy of the melting (Q_H_) or crystallisation process (Q_C_).
